# The α5 Subunit Regulates the Expression and Function of α4*-Containing Neuronal Nicotinic Acetylcholine Receptors in the Ventral-Tegmental Area

**DOI:** 10.1371/journal.pone.0068300

**Published:** 2013-07-15

**Authors:** Susmita Chatterjee, Nathan Santos, Joan Holgate, Carolina L. Haass-Koffler, F. Woodward Hopf, Viktor Kharazia, Henry Lester, Antonello Bonci, Selena E. Bartlett

**Affiliations:** 1 Translational Research Institute, Institute for Health and Biomedical Sciences, Faculty of Health, School of Clinical Sciences, Queensland University of Technology, Brisbane, Australia; 2 Ernest Gallo Clinic and Research Center at the University of California, San Francisco, Emeryville, California, United States of America; 3 Clinical Pharmacology and Experimental Therapeutics, School of Medicine, University of California, San Francisco, California, United States of America; 4 Division of Biology, California Institute of Technology, Pasadena, California, United States of America; 5 Intramural Research Program, National Institute on Drug Abuse, Baltimore, Maryland, United States of America; 6 Department of Neurology, University of California, San Francisco, California, United States of America; 7 Solomon Snyder Department of Neuroscience, Johns Hopkins University, Baltimore, Maryland, United States of America; University of Toronto, Canada

## Abstract

Human genetic association studies have shown gene variants in the α5 subunit of the neuronal nicotinic receptor (nAChR) influence both ethanol and nicotine dependence. The α5 subunit is an accessory subunit that facilitates α4* nAChRs assembly *in vitro*. However, it is unknown whether this occurs in the brain, as there are few research tools to adequately address this question. As the α4*-containing nAChRs are highly expressed in the ventral tegmental area (VTA) we assessed the molecular, functional and pharmacological roles of α5 in α4*-containing nAChRs in the VTA. We utilized transgenic mice α5+/+(α4YFP) and α5-/-(α4YFP) that allow the direct visualization and measurement of α4-YFP expression and the effect of the presence (α5+/+) and absence of α5 (-/-) subunit, as the antibodies for detecting the α4* subunits of the nAChR are not specific. We performed voltage clamp electrophysiological experiments to study baseline nicotinic currents in VTA dopaminergic neurons. We show that in the presence of the α5 subunit, the overall expression of α4 subunit is increased significantly by 60% in the VTA. Furthermore, the α5 subunit strengthens baseline nAChR currents, suggesting the increased expression of α4* nAChRs to be likely on the cell surface. While the presence of the α5 subunit blunts the desensitization of nAChRs following nicotine exposure, it does not alter the amount of ethanol potentiation of VTA dopaminergic neurons. Our data demonstrates a major regulatory role for the α5 subunit in both the maintenance of α4*-containing nAChRs expression and in modulating nicotinic currents in VTA dopaminergic neurons. Additionally, the α5α4* nAChR in VTA dopaminergic neurons regulates the effect of nicotine but not ethanol on currents. Together, the data suggest that the α5 subunit is critical for controlling the expression and functional role of a population of α4*-containing nAChRs in the VTA.

## Introduction

Neuronal nicotinic acetylcholine receptors (nAChRs) are pentameric ligand-gated ion channels with a vast diversity of subtypes [1]. The different nAChR subtypes are made up of α_2-6_ and β_2-4_ subunits in the heteromeric form or α_7-10_ subunits in the homomeric form, where each subunit is encoded by a distinct gene [2,3]. The nAChRs are abundant in several brain areas including the ventral tegmental area (VTA) [4,5], which is part of the midbrain dopaminergic reward system [6,7]. The subunit composition of nAChR is dependent on the brain region and neuronal type [8–11]. The α4β2* (*denotes the possibility that other nAChR subunits are present in the pentameric nAChR), and α7 are the most highly expressed subtype in the brain [12,13].

A wide range of pharmacological compounds have been found to activate nAChRs [14]. The neurotransmitter acetylcholine (ACh) is an endogenous agonist that can bind and activate nAChRs [15]. ACh or an exogenous agonist such as nicotine has a distinct binding site that is different from allosteric modulators such as ethanol [14]. The pharmacological, Ca^2+^ permeability and desensitization properties of these ion channels to different agonists such as ACh, nicotine or ethanol are influenced by the subunit composition of the nAChR. For example the α4β2* compared to α7 nAChRs have a slower nicotinic current kinetics with reduced Ca^2+^ ion permeability and a stronger desensitization to nicotine [16–19].

Recent human genetic association studies identified variants in the *CHRNA5* gene encoding the α5 nAChR subunit have the risk of developing ethanol or nicotine dependence [20–23]. Hence, the α5* nAChRs may be a promising target for alcohol and nicotine cessation therapy. The VTA plays a key role in the acquisition of behaviors reinforced by addictive drugs such as ethanol and nicotine [6,7], and both nicotine and ethanol can activate VTA neurons via nAChRs [24–26]. The VTA has a high concentration of the α4β2* nAChR subtype, predominantly found in dopaminergic and GABAergic neurons, and the α7 nAChRs on presynaptic glutamatergic terminals [24,27,28].

α5 is an accessory subunit that does not contribute to the formation of agonist binding site and is only co-expressed with other α and β nAChR subunits. It is present in high concentrations in the VTA, and is thought to be an important component of the putative functional (α4β2) _2_α5 nAChR subtype expressed in this region [4,29]. Cell-based heterologous expression systems have been widely used along with recent animal behavioral studies to understand α5 nAChR pharmacology. The presence of α5 subunits in α4* nAChRs produces larger nicotinic currents and modifies ACh sensitivity of α4*-containing nAChRs in cultured neurons and prefrontal cortex [30–33]. Behaviorally, the α5 nAChR subunit has been strongly associated with nicotine’s effects in rodents, since α5-/- mice display altered anxiety-related behavior [34], low sensitivity to high doses of acute nicotine [35] and increased nicotine intake at very high aversive doses [36]. Recently, it was shown that α5 nAChR subunit is important for the sedative effects of ethanol but not consumption in mice (Santos et al., 2012). However, nothing is known so far about the expression and functional contribution of α5 for nicotine and ethanol in the ventral tegmental area of the brain.

Specific nAChR subunits have been impossible to visualize and quantify expression of *in vivo* because of the lack of subtype specific tools. Here, we have developed a novel mouse line by crossing α5 nAChR deficient mice with α4-YFP nAChR knock-in (KI) mice, allowing us to directly determine the role of α5 in regulating protein expression of α4*-containing nAChRs in the brain. We found α5 to play a key role in controlling the expression of α4*-containing nAChRs in the VTA that likely affects the strength of nicotinic receptor currents of VTA dopamine neurons studied here. Additionally, the presence of α5 appears to play no additional functional role in ethanol’s effect on nAChRs in ventral tegmental area.

## Methods and Materials

### Animals and Housing

All mice were housed in climate controlled rooms with food and water available *ad libitum*. Mice were housed 2-5 per cage on a 12 hour light/dark cycle (lights on 7am).

### Ethical Considerations

The experiments contained herein comply with the laws of USA. All procedures were pre-approved by the Gallo Center ethics committee and were in accordance with NIH guidelines for the Humane Care and Use of Laboratory Animals.

### *α5* nAChR deficient mice

The α5-/- mice were generously provided by Dr. Jerry Stitzel (Institute for Behavioral Genetics, University of Colorado), and had been backcrossed at least 10 generations on a C57BL/6J background. The α5+/+ mice and α5-/- littermate mice used here were generated from heterozygous breeding pairs. The α5-deficient mice have a healthy appearance and no abnormalities in a standard battery of behavioral tests [35].

### α*4YFP*, α5+/+(α*4YFP*) *and* α5-/-(α*4YFP*) *mice*

The α4YFP knock-in mice (α4 nAChR subunit tagged with yellow fluorescent protein (YFP)) generated by the Lester Lab (Caltech) had been backcrossed on a C57BL/6J background for at least 10 generations [37]. The α4YFP mice retained the receptor function when fluorescent proteins were inserted into the intracellular M3-M4 intracellular loop of the α4 subunit. In addition, the tagged α4 nAChRs displayed similar localization patterns in the brain and are under the control of the same promoters, enhancers and trafficking mechanisms as the WT α4 [38]. Two further generations of backcrossing were performed after arrival. The mice used in this study were generated from homozygous breeding pairs. The α4-YFP mice have a healthy appearance and receptor function and have been shown to be similar to wild-type mice [38].

To be able to directly visualize and measure the contribution of α5 to α4 subunit regulation, α5+/+ and α5-/- mice were cross-bred with the α4YFP mice to create α5+/+(α4YFP) and α5-/-(α4YFP) mice. Homozygous α5-/- mice were bred with homozygous α4YFP mice to produce heterozygous α5+/- heterozygous α4YFP mice. The male and female heterozygous α5+/- and heterozygous α4YFP littermates were then mated. From these offspring heterozygous α5+/- and homozygous α4YFP littermates were mated such that all offspring produced from these pairs possessed both α4YFP genes with only the number of α5 subunit genes varying between the offspring. The α5+/+(α4YFP) mice have a healthy appearance and did not appear to be different from α5-/-(α4YFP) mice. Genotyping for α5 nAChR-deficient, α4YFP, α5+/+(α4YFP) and α5-/-(α4YFP) mice was performed using polymerase chain reaction as previously described for the α5 gene [35] and the α4YFP gene [37].

### Immunohistochemistry and Imaging

Male α5+/+(α4YFP) and α5-/-(α4YFP) mice (p35-p56 age) were deeply anesthetized with 200 mg/kg Euthasol® (Virbac, TX) and intracardially perfused with 0.9% NaCl, followed by 4% paraformaldehyde (Sigma-Aldrich, MO). Extracted brains were further fixed in 4% paraformaldehyde for 4 hours and 30% sucrose for 2 days. 50 µm frozen sections were prepared using a Microm cryostat (Thermo, Fisher Scientific, MA). Free-floating horizontal sections containing the VTA were stained with FITC-conjugated goat anti-GFP polyclonal antibody, also recognizing YFP (1:1000, ab6662, Abcam, MA) [39,40], mouse anti-Tyrosine Hydroxylase monoclonal antibody (1:2000, TH, Sigma-Aldrich, MO) followed by Alexa Fluor 594-labeled donkey anti-mouse secondary antibody (1:300, Invitrogen, CA) before mounting on slides. In addition to YFP and TH markers, we also performed triple-labeling experiments by adding a rabbit polyclonal antibody recognizing GAD65/67 (1: 500; Millipore) followed by Alexa Fluor 594-conjugated donkey anti-rabbit secondary antibody, and the TH was visualized with Alexa Fluor 647-conjugated donkey anti-mouse secondary antibody. Images were acquired using a Zeiss LSM 510 META laser confocal microscope (Zeiss MicroImaging, Thornwood, NY, US) or Nikon Eclipse Ti-E Motorized Inverted Microscope (Nikon Instruments Inc, Melville, NY). VTA images were taken in areas similar to those used for electrophysiology immediately medial to the medial terminal nucleus of the accessory optic tract (MT) in primarily the more ventral sections containing the VTA (43). Images were processed using the Imaris Neuroscience software pack (v.7.1.1, Andor Technology, Belfast, Northern Ireland); the colocalization study for the YFP protein and the TH or GABA protein was performed using ImageJ plugins (v 1.43m) (NIH).

### Western Blots

#### Preparation of homogenates

Brains were harvested from male α5+/+(α4YFP) and α5-/-(α4YFP) at p35-p70 age and 1 mm coronal sections were made using an ice cold brain matrix (Australian National University). Section(s) containing the VTA were placed on an ice cold platform and dissected under a microscope (Leica S6D, IL) and stored at -80^°^C. On the day of the analysis, VTA were thawed and then homogenized in lysis buffer (phosphate buffered saline containing 0.1% Triton-X and complete mini-protease inhibitor) with 0.5 mm glass beads using the Bullet Blender (Next Advance, NY) at 4^°^C. Protein concentration was determined using Bradford protein reagent (BioRad, CA) and the SpectroMax spectrophotometer (Molecular Devices, CA). Samples were diluted to the appropriate concentration (20 µg/lane) in reducing sample buffer (Pierce Protein Research Products, IL) and incubated at 37^°^C for 30 min.

#### Protein separation and Analysis

Proteins were separated using SDS-PAGE with 4-20% tris-glycine gels and transferred under ice cold conditions to a nitrocellulose membrane. Membranes were blocked in phosphate-buffered saline containing 5% milk and 0.05% Tween 20 then probed with primary antibodies at 4°C overnight. Rabbit polyclonal antibody against GFP (1:2500, ab290, Abcam, MA) and mouse monoclonal anti-GAPDH antibody (1:10000, MA1-22670, Affinity Bioreagents Inc, CO) were used. Appropriate Dylight 800-conjugated secondary antibodies (1:10000, Rockland Immunochemicals, PA) were used for band detection with the Odyssey Infrared Imaging System (LI-COR Biosciences, NE). Band densities were measured using Odyssey Application Software version 2.0.40 (LI-COR Biosciences, NE). An exclusion criterion was applied and α4-YFP expression levels of less than 1% of GAPDH were removed from both genotypes.

### Electrophysiology

Male mice (P21-31) were deeply anesthetized and perfused transcardially with ~ 20 ml of ice-cold modified artificial cerebrospinal fluid (aCSF): 75 sucrose; 87 NaCl, 2.5 KCl, 1.25 NaH_2_PO_4_, 7 MgCl_2_, 0.5 CaCl_2_, 25 NaHCO_3,_ saturated with 95% O_2_-5% CO_2_. Horizontal VTA brain slices (200 µm) were prepared in the same solution and recovered for at least 1hr at 31^°^C in aCSF, osmolarity 304-306, containing (in mM): 126 NaCl, 2.5 KCl, 1.1 NaH_2_PO_4_, 1.4 MgCl_2_, 2.4 CaCl_2_, 11 D-glucose and 26 NaHCO_3_ with ascorbic acid (1 mM) added just before the first slice.

Whole-cell voltage-clamp recordings made with Multiclamp 700B amplifier using Clampex 9.0 acquisition software (Molecular Devices, Sunnyvale) with acquisition rate of 10KHz and low-pass filtering at 2KHz. Experiments were performed on VTA dopaminergic neurons located immediately medial to the medial terminal nucleus (MT) of the accessory optic tract and identified by the detection of a large I_h_ current [41,42]. Recently, studies have shown that the presence of an I_h_ current does not unequivocally identify DA neurons [43]. We were consistent in our patching area where majority of I_h_ positive neurons are dopaminergic neurons (TH positive) [41,42]. Hence, it’s likely that the number of I_h_ positive TH negative neurons that contributed to this study is very small. Neurons were held at -70 mV and recordings made with 3-5 MΩ resistance patch-pipettes using a cesium-based internal solution containing: 117 mM cesium methanesulphonate, 20 mM HEPES, 0.4 mM EGTA, 2.8mM NaCl, 5 mM TEA-Cl, 2.5mg/ml Mg-ATP and 0.25 mg/ml Mg-GTP, at pH=7.2-7.4 and osmolarity 280-285. The input resistance (R_i_) and series resistance (R_s_) were continuously monitored throughout the recording and cells with any large deviations of these properties were not included in the analysis. All pharmacological experiments included atropine (1 µM) in aCSF to block muscarinic acetylcholine receptors.

Nicotinic currents were activated by pressure application of acetylcholine (ACh, 1 mM) via picospritzer pipettes (10 psi, Parker Hannifin Instrument, Cleveland, OH) (adapted from [18]). Neurons with stable holding current for 5 min were puffed with ACh estimated to be ~20 µM from the neuron for 300 ms every 2 min for 6 min. The average of the three peak inward currents (evoked every 2 min across 6 min) was taken to be the baseline was calculated relative to the holding current 500 ms immediately before the ACh puff using Clampfit 9.0 acquisition software (Molecular Devices, Sunnyvale, CA). A drug was then bath applied for 10 min during which time ACh was puffed every 2 min followed by 10 min wash-out period. The amplitude of the ACh-induced current at each time point was measured as percent change of baseline current induced by the drug: [(amplitude of ACh-induced current at x min)) / amplitude of ACh-induced baseline current] X 100. The drugs used here were nicotine (0.3 µM and 1 µM), ethanol (60 mM and 80 mM), dihydro-β-erythroidine (DHβE, 2 µM), methyllycaconitine (MLA, 5 nM) or tetrodotoxin (2 µM). Only one drug concentration was applied per neuron. We observed a low incidence of fast nAChR currents which could be because of not using a computer-controlled motorized puffer that could be retracted after puffing. Hence, any agonist leakage could potentially cause a loss or underestimate the fast component elicited by mainly the α7 nAChR [18].

### Drugs

The 95% (v/v) ethanol (Gold Shield Chemical Co, CA), nicotine hydrogen tartrate, atropine, DHβE, MLA, tetrodotoxin, acetylcholine chloride (Sigma-Aldrich, MO) solutions were prepared fresh daily for all experiments.

### Statistics

We used Graph Pad Prism (Graph Pad, CA) or Sigma Stat (Systat Software, CA), using two-way, one-way ANOVA or un-paired t-test wherever applicable with Newman–Keuls post hoc analysis when a significant effect was found (p < 0.05).

## Results

### α5 subunits help maintain the expression of α4*-containing nAChR in the VTA

The α4*-containing nAChRs are highly expressed in the VTA [44]. The α5 functions as an accessory subunit and assembles predominantly with the α4*-containing nAChRs in the VTA [1,29]. We wanted to first examine whether the presence of α5 is critical for maintaining VTA α4 protein levels. Since visualization and quantification of nAChRs has been difficult due to lack of specific antibodies; we utilized transgenic mice in which the α4 subunit of nAChRs is fused with yellow fluorescent protein (YFP) to which available specific antibodies can be effectively used in western blot analysis to quantify α4 protein levels. To assess the role of the α5 nAChR subunit in regulating α4 protein levels, we crossed α4YFP knock-in mice with the α5 knockout mice to generate α5+/+(α4YFP) and α5-/-(α4YFP) mice (*see Materials and Methods*). These mice were normal in their weight, appearance and showed no obvious signs of physical or neurobiological deficits. They had good fertility and produced expected proportions of transgenic mice from mating and were viable.

**Figure 1:**
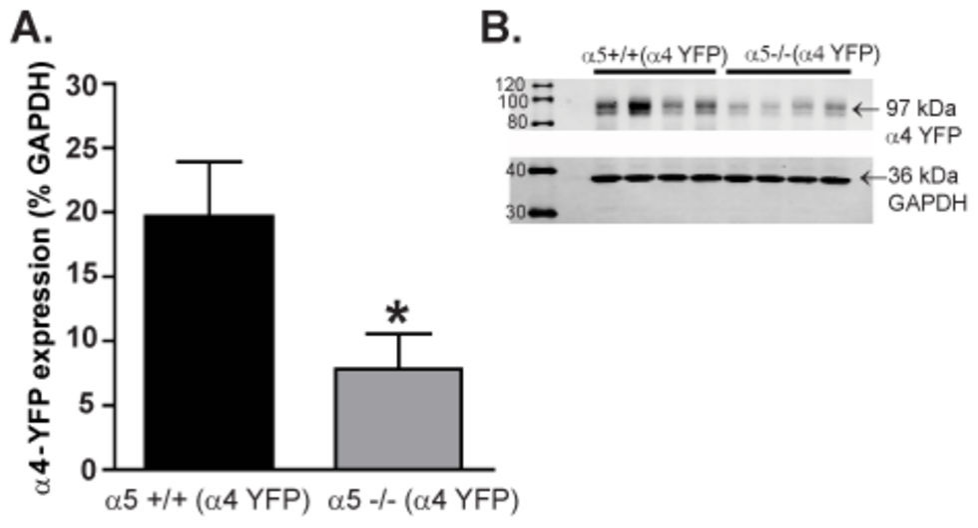
The α5 subunit plays an importantrole in maintaining α4* nAChR levels. (A and B) The α5-/-(α4 YFP) mice have significantly reduced α4YFP expression levels in the VTA compared with α5+/+(α4 YFP) quantified using western blot analysis. The values are expressed as mean α4 YFP expression (% of GAPDH) ± SEM (two-tailed unpaired t-test, * p=0.05). n=6-8 number of animals (two-tailed unpaired t-test, *p<0.05).

Using anti-GFP(YFP) antibodies in western blot analysis, we found that the α4YFP expression measured in VTA tissue sections (*see Materials and Methods*) was significantly reduced in the α5-/-(α4YFP) (7.84 ± 2.7, n=6 animals) when compared to α5+/+(α4YFP) mice (19.67 ± 4.2, n=8 animals) (two-tailed unpaired t test, *p<0.05, Figure 1A& B). Hence, the absence of α5 causes a substantial reduction in the α4 subunit expression in the total tissue homogenates of the VTA.

**Figure 2:**
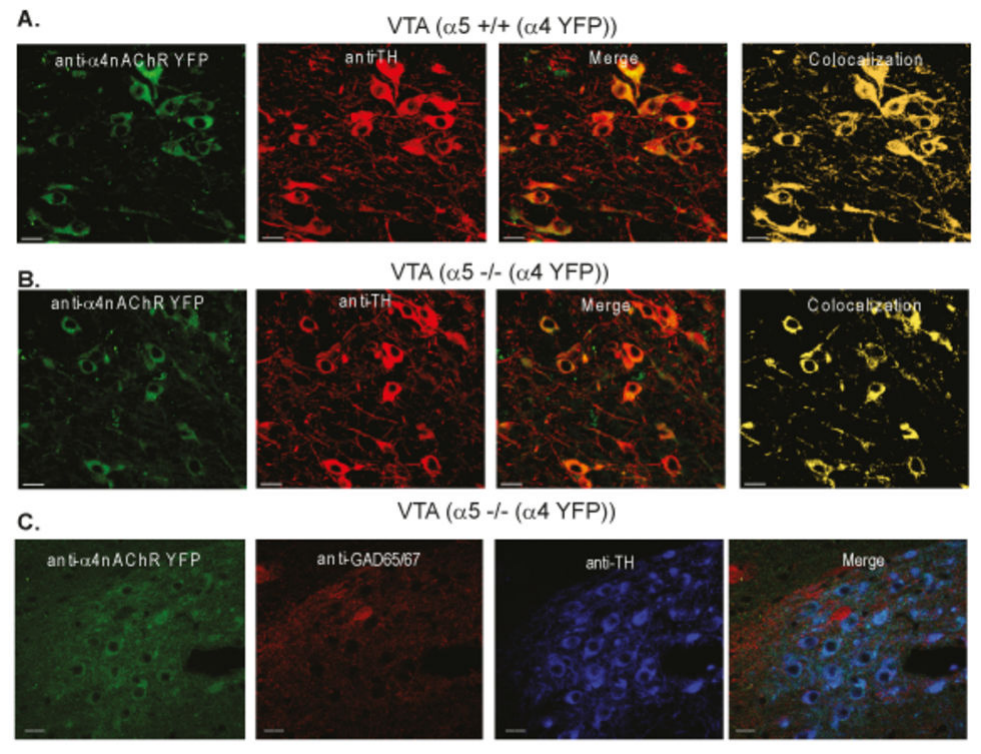
The α4 nAChR is colocalized with TH-positive dopaminergic neurons of the VTA. Representative immunofluorescence images from α5+/+(α4 YFP) (A) and α5-/-(α4 YFP) (B and C); VTA showing α4 nAChR-YFP expression (green), tyrosine hydroxylase (TH) (red) expression, the merged images (green + red) and the colocalization (yellow); VTA showing α4 nAChR-YFP expression (green), GAD65/67 (red), tyrosine hydroxylase (TH) (blue) expression, and the merged images (green + red + blue). Scale bar is 30µm.

The α4* nAChRs are found in both dopaminergic and GABAergic neurons of the VTA [44,45]. Semi-quantitative colocalization analysis of the VTA that correspond to areas where we performed electrophysiology in α5+/+(α4YFP) and α5-/-(α4YFP) mice showed that α4YFP is co-expressed in the majority of TH-positive dopaminergic neurons in both genotypes (Figure 2A& B) (α5+/+(α4YFP): n=2 animals; α5-/-(α4YFP): n=3 animals). Triple-staining with antibodies against GFP (YFP), GAD65/67 (GABAergic marker) and TH shows that GAD65/67-positive perikarya express much less YFP than adjacent TH-positive dopaminergic cells which extend the data previously described by (Nashmi et al., 2007) (38) (Figure 2C).

### α5 subunits enhance the strength of α4* nicotinic currents in VTA dopaminergic neurons

**Figure 3:**
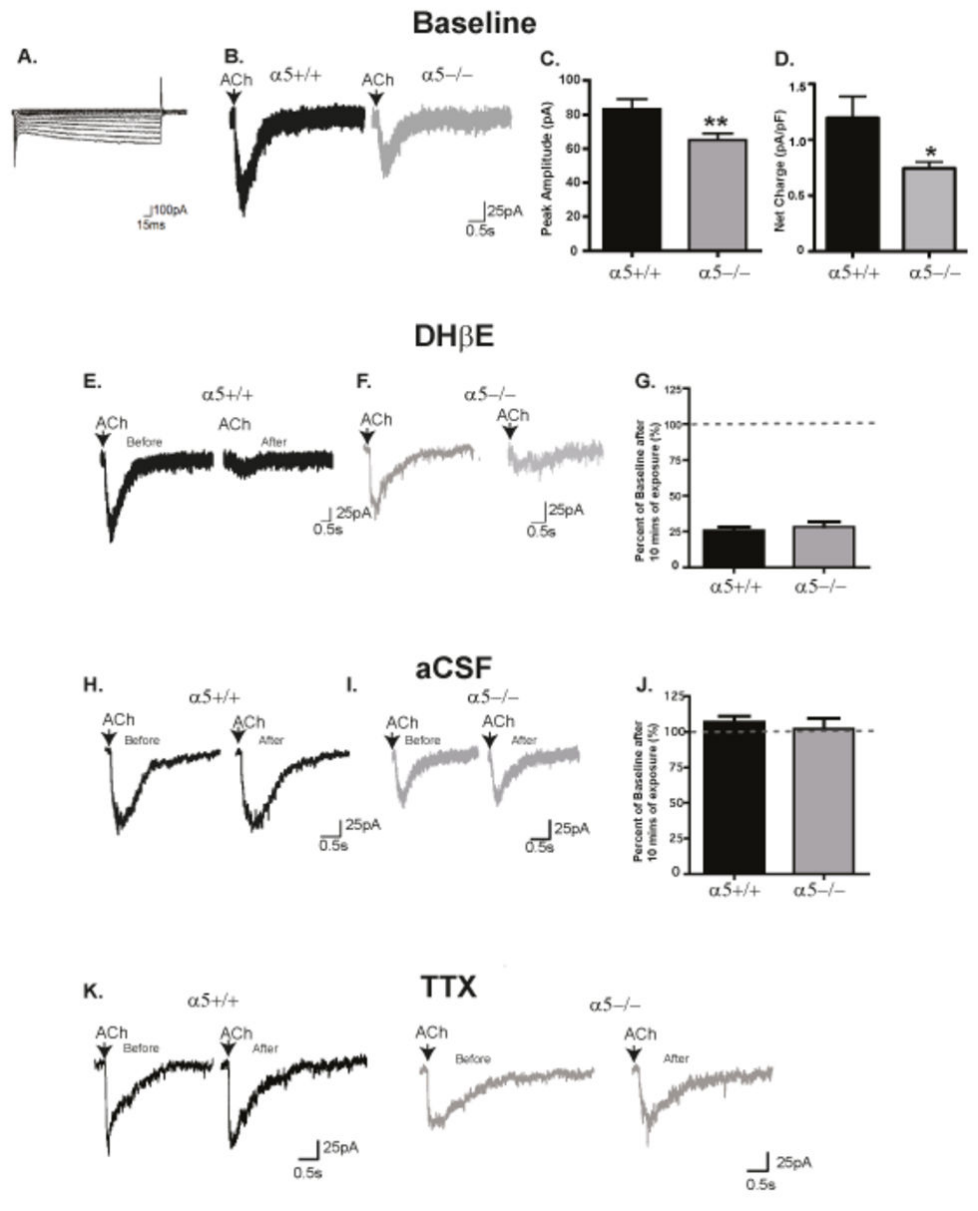
The α5 subunit controls the strength of nicotinic currents mediated by the α4*-containing nAChRs in VTA dopaminergic neurons. (A) A typical I_h_ current. (B) Sample voltage clamp traces of peak inward current of DA neurons to a 300 ms ACh (1mM) puff in α5+/+ (black) and α5-/- mice (gray). (C) The average ACh-induced peak current amplitude was reduced in dopaminergic neurons from α5-/- mice in comparison to α5+/+ mice. (D) The average net charge was reduced in dopaminergic neurons from α5-/- mice in comparison to α5+/+ mice. (E and F) Both α5+/+ (black) (E) and α5-/- (gray) (F) mice showed a nearly complete reduction in the nicotinic currents after 10 min of α4 nAChR antagonist DHβE (2µM) treatment indicating that the responses are mediated by the α4* nAChRs. (G) The percent reduction from baseline following DHβE treatment were similar for α5+/+ and α5-/- mice. (G and H) Currents were stable to 300ms ACh puffing every 2 min for 20 min in neurons exposed to aCSF in both α5+/+ (black) (H) and α5-/- (gray) (I). (J) There was no significant percent reduction from baseline in both genotypes. (K) TTX (2 µM) had no effect on the current in both α5+/+ and α5-/- mice. In C & D, n = 57-61 cells across 45-50 animals, F, n =7 cells across 6 animals and in I, n=6 cells across 5-6 animals. The values in C are mean peak amplitude ±SEM (two-tailed unpaired t-test, **p<0.01). The values in F&I are reported as mean percent of baseline ±SEM (two-tailed unpaired t-test). The calibrations for the current trace are 100pA, 15 sec (A) and 25pA, 0.5sec (B, E and H).

To assess the functional effect of reduced α4 protein levels, we examined here the nAChR activation of dopaminergic neurons in VTA brain slices from α5+/+ and α5-/- mice. Patch experiments were performed in neurons near the medial terminal nucleus of the accessory optic tract (MT), where the Ih current typically indentifies dopaminergic neurons in mice [41,46]; putative dopaminergic neurons were thus identified by the presence of an Ih current [18,19,42] Figure 3A). We performed whole-cell voltage clamp recordings at -70 mV, and nAChR currents were elicited by puff application of ACh (1 mM, 300 ms, applied every 2 min) (Figure 3B) in the presence of atropine (1 µM) to block muscarinic acetylcholine receptors. We found that the peak amplitude of the nicotinic current elicited by ACh was significantly smaller in α5-/- neurons (65.1 ± 3.7 pA, n=61 cells across 50 animals) compared to α5+/+ neurons (83.2 ±5.8 pA, n=57 cells across 45 animals) neurons (two-tailed unpaired t test, **p<0.01, Figure 3C). We found no difference between the capacitance value of these cells between α5+/+ and α5-/-mice (α5+/+: 77.85 ± 5.4; α5-/-: 72.64 ±3.8). However the net charge (pA/pF) calculated for these neuronal cells also determined a significant difference (α5+/+: 1.196 ± 0.1938; α5-/-: 0.7455 ± 0.05617; two-tailed unpaired t test, *p<0.05, Figure 3D).

Importantly, almost all evoked nAChR currents were sensitive to the α4* nAChR antagonist dihydro-β-erythroidine (DHβE) (2 µM, 10 min, n=4-6 per genotype, Figure 3 E& F); the percent of baseline current following DHβE application was 25.6 ± 2.5% (n=7 cells across 6 animals) for α5+/+ and 28.4 ± 3.4% (n=7 cells across 6 animals) for α5-/- mice (Figure 3G) (two-tailed unpaired t test, n.s). This confirmed that the ACh-evoked current predominantly reflected α4*-containing receptors, and that α4* currents were reduced in the absence of α5 subunits. Currents with a fast component [18] were rarely observed, and were inhibited by the α7 nAChR antagonist MLA (5 nM, 10 min) in both genotypes (data not shown). In addition, ACh-evoked currents were not reduced by the sodium channel blocker tetrodotoxin (2 µM, 10 min, n=3-5 per genotype, Figure 3K), suggesting that ACh-evoked currents did not reflect changes in presynaptic release and instead represented postsynaptically-evoked nAChR-mediated currents. Finally, repeated ACh puffing led to currents that were stable in amplitude for >20 min in neurons exposed only to aCSF in both α5+/+ (Figure 3H) and α5-/- (Figure 3I), suggesting that this method could reliably be used in subsequent experiments examining changes in nAChR currents with exposure to ethanol and nicotine. The percent of baseline current following 20 min puffing ACh in the presence of aCSF was 106.6 ± 4.3% (n=6 cells across 5 animals) for α5+/+ and 102 ± 7.4% (n=6 cells across 6 animals) for α5-/- mice (Figure 3J) (two-tailed unpaired t test, n.s).

### α5α4* nAChR subunits reduce receptor desensitization during nicotine exposure

**Figure 4:**
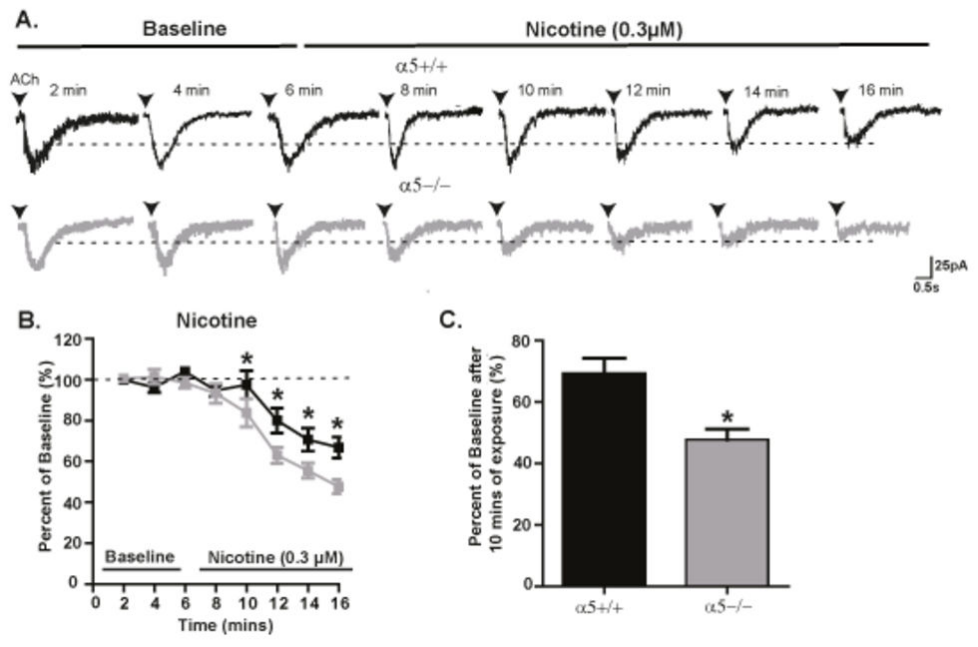
The presence of α5α4* nAChR protects receptors against desensitization to nicotine exposure. (A) The amplitude of a 300 ms ACh-induced sample current trace elicited every 2 min during a 10 min exposure to nicotine (0.3 µM) is reduced from baseline in neurons from both α5+/+ (black) and α5-/- (gray) mice. (B) The time course of the reduction of current from baseline for a 10 min exposure of 0.3 µM nicotine in α5+/+ and α5-/- mice. (C) The average percent of baseline current after 10 min of 0.3 µM nicotine exposure. In B&C, n=8-10 cells across 8-9 animals. The values in B&C are reported as mean percent of baseline ± SEM (two-way ANOVA followed by Neuman-Keuls post hoc test, *p<0.05). The calibrations for the current trace are 25 pA, 0.5 sec.

It is well known that nAChRs undergo desensitization, a reversible reduction in current response with prolonged application of an agonist such as nicotine [19,32], including in VTA dopaminergic neurons [19]. Here, we examined the effect of bath application of nicotine (0.3 µM and 1 µM, 10 min) on ACh-induced currents in VTA dopaminergic neurons in slices taken from α5+/+ and α5-/- mice. The average of three ACh-induced current responses (evoked every 2 min across 6 min) was considered as baseline, and responses in the presence of nicotine represented as percent of baseline (*see Materials and Methods*). Continued exposure to nicotine reduced the amplitude of ACh-induced currents (Figure 4A) in both the α5+/+ and α5-/- neurons, and nAChR desensitization was significantly greater in the absence of the α5 subunit (Figure 4B). Two-way ANOVA analysis of percent reduction from baseline for 0.3 µM nicotine revealed a significant effect of genotype (F(1,128) = 14.05, p<0.001), exposure time (F(7,128) = 32.66, p<0.001) and genotype-time interaction (F(7,128) = 2.15, p<0.05), with post-hoc analysis indicating a greater reduction in α5-/- versus α5+/+ at 10-16 min (Figure 4B). The percent baseline current following 10 min application of 0.3 µM nicotine was 69.2 ± 5% (n=9 cells across 9 animals) for α5+/+ and 47.7 ± 3.4 (n=8 cells across 8 animals) for α5-/- mice (Figure 4C). For the 1 µM dose of nicotine, two-way ANOVA analysis revealed a significant effect of time (F(7,159) = 71.07, p<0.001) and genotype (F(1,159) = 4.13, p<0.05) but no effect on genotype-time interaction (F(7,159) = 1.41, n.s) (α5+/+: 49.9±3.7%, n=10 cells across 9 animals; α5-/-: 36.7±3.8%, n=9 cells across 8 animals).

### α5α4* nAChR does not affect ethanol-mediated potentiation of ACh-induced nicotinic current

**Figure 5:**
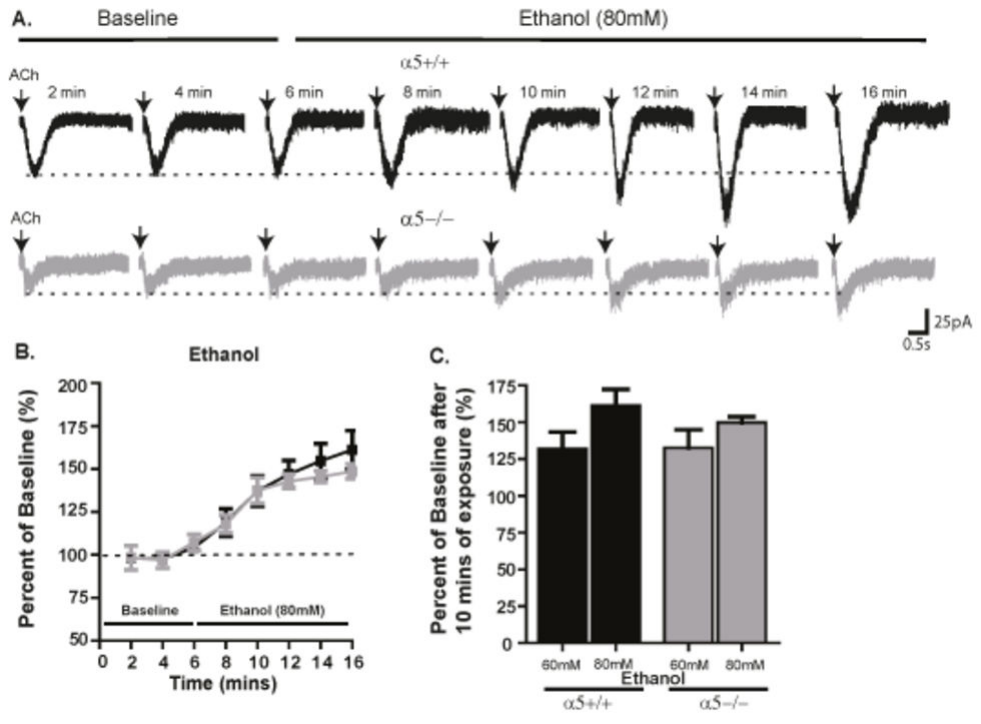
The presence of α5α4* nAChR does not affect ethanol-mediated potentiation of ACh-induced nicotinic current. (A) The amplitude of a 300 ms ACh-induced sample current trace elicited every 2 min during a 10 min exposure to ethanol (80 mM) is increased from baseline in neurons from α5+/+ (black) and α5-/- (gray) mice. (B) The time course of the potentiation of current from baseline for a 10 min exposure of 80mM ethanol in α5+/+ and α5-/- mice. (C) The average percent of baseline current after 10 min of 60 mM and 80 mM ethanol exposure for α5+/+ and α5-/- mice. In B&C, n= 5-10 cells across 5-8 animals. The values in B&C are reported as mean percent of baseline ± SEM (two-way ANOVA followed by Neuman-Keuls post hoc test). The calibrations for the current trace are 25 pA, 0.5 sec.

Ethanol has been shown to potentiate ACh-induced nicotinic currents in cultured neurons [15]. To the best of our knowledge, we demonstrate for the first time that ethanol (60 mM and 80 mM, Figure 5A) can significantly increase the amplitude of ACh-induced currents in VTA dopaminergic neurons of α5+/+ and α5-/- mice. We found no difference in the level of ethanol-induced potentiation of ACh-induced currents in the absence or presence of α5 subunit. A two-way ANOVA revealed a significant effect of 80 mM ethanol exposure time (F(7,128) = 24.96, p<0.001) but no effect of genotype (F(1,128) = 0.77, n.s) or genotype-time interaction (F(7,128) =0.319, n.s). Post hoc analysis revealed no significant effect (Figure 5B). Similarly, a two-way ANOVA analysis revealed a significant effect of 60 mM ethanol exposure time (F(7,64) = 11.97, p < 0.001) but no effect of genotype (F(1,64) = 1.707, n.s) or genotype-time interaction (F(7,64) = 0.55, n.s) (Figure 5C). The percent of baseline current following 10 min application of 80 mM ethanol was 161.2 ± 11.1% (n=10 cells across 8 animals) for α5+/+ and 149.8 ± 4% (n=8 cells across 6 animals) for α5-/- mice and, for 60mM ethanol application, was 131.8 ± 11.4% (n=7 cells across 5 animals) for α5+/+ and 132.6 ± 12.3% (n=8 cells across 5 animals) for α5-/- mice (Figure 5C).

## Discussion

The α4β2* nAChR is widely expressed in the brain and within the ventral tegmental area (VTA) the α5 is an accessory subunit expressed predominantly in (α4β2) _2_α5 nAChRs [44,47,48]. There is considerable evidence in *in vitro* cell-based systems that the inclusion of α5 subunit can regulate the pharmacological properties, Ca^2+^ permeability and ACh sensitivity of α4β2 nAChR cell lines [30,31,33,49]. Our study is the first *ex vivo* evidence to show that the α5 nAChR subunit controls α4*-containing nAChR expression in the ventral tegmental area (VTA).

The first level of regulating nAChR expression is the transcription of the subunits. The α5-/- mice were found to have normal transcript levels for all nAChRs subunits, including α4 and β2 in all brain areas including the VTA [35,50]. Although in midbrain dopamine neurons, there is no modulation of α4 and β2 mRNA from birth through adulthood [51], there is a transient increase in α5 mRNA shortly after birth (~p20) which declines through adulthood. In studies involving cell-lines expressed in oocytes, the subunit compositions of nAChRs expressed on the cell surface are dependent on the relative proportions of subunits (cDNAs) available for assembly [52,53]. The inclusion of α5 subunit in the pool with α4 and β2 was shown to increase the number of high binding affinity site measured by [^3^H] epibatidine in HEK cells compared to the α4β2 parent line [31]. Hence it may be possible that the postnatal surge in α5 mRNA could be facilitating the increase in α4*-containing nAChRs in the VTA of α5+/+ mice. Because of the lack of α5 mRNA in the knockout mice, the number of α4*-containing nAChRs is reduced. This is how α5 may influence the assembly of α4*-containing nAChRs in the VTA. The reduced α4 protein levels measured here could be at the surface or intracellular or both. Hence determining if this regulation of α4 nAChR subunit expression has key implication for cholinergic function in the ventral tegmental area becomes important.

We find that greater number of α4*-containing nAChRs in the presence of α5 strengthens nicotinic receptor currents in VTA dopaminergic neurons. Nicotinic currents in both α5 +/+ and α5 -/- mice were almost fully inhibited by the α4* nAChR antagonist DHβE, suggesting that the ACh-induced nAChR currents in VTA dopaminergic neurons were predominantly mediated by α4*-containing nAChRs, and that the presence of the α5 subunit in the α4* nAChR assembly boosted receptor currents. A caveat in our study is that electrophysiological recordings were done in animals between postnatal p21-p28 and the western blot analysis was done in animals between p35-p70. However previous studies indicate it is unlikely that there would be any difference in the expression or current between these two age groups [51,54]. The stronger current in the presence of α5 is consistent with studies involving non-neuronal cell lines where the coexpression of α5 with α4β2 nAChRs produced larger currents than α4β2 alone [30]. Additionally, one brain slice recording study in the prefrontal cortex showed increases in the amplitude of nicotinic receptor currents in cortical neurons of α5+/+ mice compared to α5-/- mice [32]. Studies from heterologous cell line suggest that the inclusion of α5 with α4β2 yield larger currents because of the formation of higher conductance channel with greater Ca^2+^ permeability [30,49]. Our observation of a critical role for α5 in maintaining expression of VTA α4* receptors suggests that the reduced strength of the nicotinic current in the absence of α5 is likely due to a reduced α4* nAChRs protein levels on the cell surface. However further molecular studies would be required to validate surface expression change. One of the functions of increased Ca^2+^ permeability through nAChR is thought to increase the excitability of the neuron and modulate neurotransmitter release [55]. Hence, the reduced nicotinic current in dopamine neurons is likely to affect excitability in the α5-/- mice.

The α5 subunit is clearly an important accessory component of the α4* nAChR assembly in the brain. Moreover, human genetic association studies have indicated that the minor allele of rs16969968 in CHRNA5, encoding a single nucleotide polymorphism in the α5 subunit of the nAChR, to be associated with increased risk of nicotine dependence [21,56] and association with the level of alcohol response to an alcohol challenge and dependence [20,23].

The human genetic studies have been complemented well with behavioral animal studies to show that α5*-containing nAChRs are important for nicotine [35,36,57]. Additionally, previous studies have shown the α4*-containing nAChRs to be important for the reinforcing properties of nicotine [24,58]. Nicotine can increase the release of dopamine neurotransmitter in the striatum facilitating the reward-related dopamine signal [59,60]. *In vitro* studies have shown nicotine at high concentrations (or prolonged exposure at low concentrations) can cause desensitization of nAChRs on dopaminergic neurons [19,61] and thereby regulating striatal dopamine release [62]. We found that prolonged exposure to nicotine at concentrations achieved by smokers [19,63] induces desensitization of nAChRs on VTA dopaminergic neurons, which is significantly enhanced in the absence of the α5 subunit. This increased nAChR desensitization in the VTA dopaminergic neurons likely reduces sensitivity to nicotine and decreases striatal dopaminergic release, which could explain the reduced sensitivity to high doses of nicotine [35] and increased nicotine self-administration [36] in α5-/- mice. These results about the α5-/- nicotinic receptors become particularly relevant in understanding the role of CHRNA5 polymorphisms for nicotine dependence in humans [64–66].

The behavioral role of α5 in ethanol’s effect has been shown to modulate the sedative effects but not ethanol consumption in mice [67]. Previous studies have shown ethanol-induced activation of the VTA DA neurons *in vivo* and during *in vitro* brain slice electrophysiology [6,68]. The interaction of ethanol with the nAChR ion channel was first demonstrated in Torpedo nAChRs, where ethanol enhances binding affinity of ACh to this receptor [69]. Ethanol can potentiate the currents evoked by ACh in cultured cortical neuronal cells [15,70] and *Xenopus* oocytes expressing different subunit compositions nAChRs [16,71]. Here, we observed that ethanol potentiates ACh-induced nicotinic currents in slice recording from the VTA, with similar potentiation in neurons from both α5+/+ and α5-/- mice. Hence, the α4*-containing nAChRs participated in ethanol’s potentiation of ACh-evoked current irrespective of the α5 subunit. To the best of our knowledge, this is also the first report of ethanol’s effect on ACh evoked currents in the dopaminergic neurons of the VTA, consistent with oocyte studies showing that α4β2 nAChRs were potentiated with ethanol (75mM) [16,17]. Together, the α5* nAChRs appear to play a key role in the pharmacology of nicotine but not ethanol modulation of nicotinic currents in VTA dopaminergic neurons.

Our observation that α4α5* nAChR appear to not play a regulatory role in ethanol’s effect is not completely surprising. In behaving animals studies using null mutant mice of the β2 nAChR subunits [72], α4 nAChR subunits [73] and α5 nAChR subunits [67] found no role in baseline ethanol consumption. In addition, pharmacological manipulation using the α4* nAChR antagonist DHβE showed no effect on ethanol intake [74]. Moreover, recent studies indicate the α3β4* rather than the α4β2* nAChRs may play an important role in regulating ethanol consumption [75]. Although most α5 is likely associated with the α4 subunits, there is also some evidence that the α5 subunit may also be present in α3β4* nAChRs [76], which can also modulate desensitization, pharmacology, Ca^2+^ permeability of human neuronal α3* nAChRs in recombinant assays and non-neuronal expression systems [76,77]. Nonetheless, most studies indicate a prominent association of α5 subunits with the α4β2* complex [31,44,47].

In conclusion, we have shown the α5 subunit is critical for maintaining the expression of α4* nAChR protein levels of the VTA neurons and strengthening nicotinic currents in dopaminergic neurons. The presence of α5 causes resistance to nicotine desensitization but does not regulate ethanol enhancement of ACh currents in VTA dopaminergic neurons. The α5 nAChR subunit is an important component of the α4* containing nAChRs and plays a vital role for nicotine’s effect in the brain. The α5α4* nAChR appears to be a promising target for at least the treatment for nicotine dependence.
